# The standardized extract of *Centella asiatica* L. Urb attenuates the convulsant effect induced by lithium/pilocarpine without affecting biochemical and haematological parameters in rats

**DOI:** 10.1186/s12906-023-04179-2

**Published:** 2023-09-27

**Authors:** Eduardo Rivadeneyra-Domínguez, Isaac Zamora-Bello, Juan Manuel Castañeda-Morales, Joel Jahaziel Díaz-Vallejo, Óscar Rosales-Sánchez, Juan Francisco Rodríguez-Landa

**Affiliations:** 1 Facultad de Química Farmacéutica Biológica, Universidad Veracruzana, Xalapa, Veracruz México; 2 Instituto de Neuroetología, Universidad Veracruzana, Xalapa, Veracruz México; 3 Laboratorio de Neurofarmacología, Instituto de Neuroetología, Universidad Veracruzana, Xalapa, Veracruz México

**Keywords:** Epilepsy, *Centella asiatica*, Blood cytometry, Liver function, Renal function

## Abstract

**Background:**

Status epilepticus (SE) is a type of epileptic activity characterized by a failure of the inhibitory mechanisms that limit seizures, which are mainly regulated by the GABAergic system. This imbalance increases glutamatergic neurotransmission and consequently produces epileptic activity. It is also associated with oxidative stress due to an imbalance between reactive oxygen species (ROS) and antioxidant defences. Unfortunately, long-term treatment with anti-epileptic drugs (AEDs) may produce hepatotoxicity, nephrotoxicity, and haematological alterations. In this way, some secondary metabolites of plants have been used to ameliorate the deterioration of nervous system disorders through their antioxidant properties, in addition to their anticonvulsant effects. An example is *Centella asiatica*, a plant noted to have a reputed neuroprotective effect related to its antioxidant activity. However, similar to conventional drugs, natural molecules may produce side effects when consumed in high doses, which could occur with *Centella asiatica*. Therefore, we aimed to evaluate the effect of a standardized extract of *Centella asiatica* L. Urb with tested anticonvulsant activity on biochemical and haematological parameters in rats subjected to lithium/pilocarpine-induced seizures.

**Methods:**

Twenty-eight adult male Wistar rats were randomly divided into four groups (n = 7 each): vehicle (purified water), *Centella asiatica* (200 and 400 mg/kg), and carbamazepine (CBZ) (300 mg/kg) as a pharmacological control of anticonvulsant activity. Treatments were administered orally every 24 h for 35 consecutive days. On Day 36, SE was induced using the lithium/pilocarpine model (3 mEq/kg, i.p. and 30 mg/kg s.c., respectively), and the behavioural and biochemical effects were evaluated.

**Results:**

*Centella asiatica* 400 mg/kg increased the latency to the first generalized seizure and SE onset and significantly reduced the time to the first generalized seizure compared to values in the vehicle group. Biochemical parameters, i.e., haematic cytometry, blood chemistry, and liver function tests, showed no significant differences among the different treatments.

**Conclusion:**

The dose of *Centella asiatica* that produces anticonvulsant activity in the lithium/pilocarpine model devoid of hepatotoxicity, nephrotoxicity, and alterations in haematological parameters suggests that the standardized extract of this plant could be of utility in the development of new safe therapies for the treatment of convulsions associated with epilepsy.

## Introduction

Epilepsy is a chronic neurological disease characterized by recurrent and spontaneous seizures. The World Health Organization estimates that approximately 50 million people suffer from epilepsy [1–6]. The treatment of epilepsy is mainly focused on the use of antiepileptic drugs (AEDs), which aim to prevent seizures and restore neuronal function. Special attention has been given to preclinical research on the antioxidant effects of natural compounds on neuronal function in various brain structures, such as the hippocampus, substantia nigra, and striatum [7, 8]. For example, turmeric has been shown to reduce oxidative stress and the expression of proteins associated with apoptotic changes in the hippocampus of rats with pilocarpine-induced status epilepticus [8]. *Centella asiatica* administered to rats with parkinsonism showed a decrease in dopaminergic neuronal death in certain brain regions [7]. These neuroprotective substances play a crucial role in preventing neuronal damage caused by seizures and preserving cognitive function [9]. Diverse antioxidant substances reduce reactive oxygen species (ROS) levels and oxidative stress [10, 11]. In animal models of epilepsy, reduced oxidative stress has been associated with decreased neuronal damage and improved cognitive function [12–14]. In addition, certain anticonvulsant drugs, such as topiramate and lacosamide, possess antioxidant properties, which contribute to their therapeutic effects [15]. Moreover, some of the most widely studied natural compounds are curcumin [16], fisetin, resveratrol [17], quercetin [18, 19], and asiatic acid [20, 21], all of which exhibit antioxidant and neuroprotective effects. In this context, *Centella asiatica* has been studied in preclinical models of seizures induced by pentylenetetrazol, a noncompetitive GABA_A_ receptor antagonist, and has shown anticonvulsant potential [22–29]. These properties are attributed to asiatic acid, a triterpene with antioxidant activity [30–33].

It is important to note that the consumption of natural products can have long-term side effects and toxicity. Excessive consumption of *Camellia sinensis* (green tea) and *Piper methysticum* (Kava) has been associated with hepatocellular injury [34, 35]. The consumption of *Camellia sinensis* and *Piper methysticum* has been associated with increased levels of transaminases, alkaline phosphatase, and bilirubin in adults [36, 37]. These findings underline the need for a thorough assessment of the effects on liver and kidney function of standardized extracts of *Centella asiatica*.

## Methods

### Animals

Twenty-eight adults male Wistar rats were randomly assigned to four groups (n = 7 rats per group). The rats were housed in acrylic boxes (44 cm × 33 cm base, and 25 cm high) in the vivarium of the Laboratory of Pharmacotoxicology, Facultad de Química Farmacéutica Biológica - Xalapa, at room temperature with a light/dark cycle of 12 × 12 h (lights were turned on at 7:00 am). The average ambient temperature was 25 °C ± 2 °C. The rats had free access to food and water. At the beginning of the experiment rats weighed an average of 250 g, while at the end of the experiments they obtained an average of 380 g, approximately.

All experimental rats included in the present study were obtained from a local breeding rat stock. At 21 days of age, the rats were weaned and maintained in the vivarium until starting the experiments. Under these housing conditions, the rats were habituated to the environmental conditions.

### Dose selection

The doses of *Centella asiatica* (200 and 400 mg/kg) were based on a previous study in which it was reported that 200 mg/kg of the standardized extract of *Centella asiatica* produces anticonvulsant effects in an experimental model of epilepsy [32]. However, to explore the effect of higher doses, twice that dose was included (400 mg/kg). Before SE, CBZ 300 mg/kg every 48 h was administered for 5 alternate days, as previously reported [38–41], which was included as a positive control of anticonvulsant activity.

The doses of *Centella asiatica* (200 and 400 mg/kg) were adjusted to use 2 ml/kg body weight as the standard volume, thus standardizing the volume administered to the animals. For oral administration to the rats, the methodology used by Sharp-Villano was followed [42]. It is important to note that *Centella asiatica* and vehicle doses were administered for 35 consecutive days every 24 h. CBZ administration (pharmacological control) was performed every 48 h for 5 alternate days to avoid liver damage, as previously reported by Morrisett et al. [40] and Grabenstatter [41]. At the end of the treatment, the rats were subjected to lithium/pilocarpine-induced seizures, and behavioural and biochemical variables were evaluated.

### Experimental groups and manipulations

The 28 rats used for this study were randomly distributed into four groups (n = 7 rats per group) using free software available online (https://random.org, accessed on 14 November 2022): vehicle, *Centella asiatica* (200 mg/kg and 400 mg/kg), and CBZ groups (300 mg/kg). Twenty-four hours after the last administration of the treatments, all experimental subjects fasted for 8 h before undergoing SE induction with lithium/pilocarpine, and a behavioural assessment of SE was performed. Once the behavioural test was concluded, the animals were subjected to deep anaesthesia using an overdose of sodium pentobarbital (100 mg/kg, i.p.; Cheminova de México, Mexico City, Mexico; Reg. SAGARPA Q-7048-044) until reaching an unconscious and pain-free state, with an absence of the palpebral reflex response and paw and tail pad pinch response. Once deep anaesthesia was verified, an intracardial puncture was then performed to obtain a blood sample using a 5-ml syringe with a 22 Gx 32-mm needle. Blood was deposited in both dry tubes (without anticoagulant) and in tubes with ethylenediaminetetraacetic anticoagulant (EDTA; BD Vacutainer, Mexico City, Mexico), and subsequently, biochemical and haematological analyses were performed. Finally, the death produced by pentobarbital overdose was verified, and then the rat was deposited according to official guidelines for the handling of biological and infectious waste [43, 44].

### Status epilepticus induced by lithium-pilocarpine

On Day 35, the animals were injected with lithium chloride (LiCl, 3 mEq/kg, i.p.). Twenty hours later, pilocarpine hydrochloride (30 mg/kg, s.c.) was administered to generate SE [40, 41]. The behavioural manifestation of SE seizures was monitored according to the Racine scale, which consists of phase 0, behavioural arrest; phase I, facial myoclonus (winking ipsilateral to the site of stimulation and/or chewing); phase II, phase I behaviours and head myoclonus (repeated head downwards tilt, nodding); phase III, phase II behaviours and forelimb myoclonus; phase IV, all of the above behaviours and kangaroo posture; phase V, phase IV behaviours, loss of postural tone and drooping; and phase V, phase IV behaviours, loss of postural tone and drooping. On the other hand, latency refers to the time it takes for the first generalized seizure to occur and status epilepticus to be established after pilocarpine administration [45, 46]. Behavioural observation was performed for 60 min.

### Blood sample processing

The samples used to determine liver (albumin, ALT, AST, direct bilirubin, indirect bilirubin, total bilirubin, alkaline phosphatase, Ɣ-glutamyltransferase, and total protein) and kidney (glucose, urea, creatinine, and BUN) function were placed in Vacutainer tubes without anticoagulant, and once they coagulated, they were centrifuged at 3500 revolutions per minute to obtain the serum. Then, the serum was transferred to the dry chemistry containers found in the Vitros 250 equipment (Johnson and Johnson, Ramsey, MN, USA). Samples for haematic cytometry (MCHC, erythrocytes, Hto, Hb, MCH, leukocytes, platelets, and MCV) were collected in EDTA Vacutainer tubes and processed on the Advia 560 automated analyser (Siemens Healthcare Diagnostics Inc., Tarrytown, N.Y. USA). The test results were analysed and compared with the corresponding biological reference ranges for the Wistar rat to detect any alterations at the hepatic, renal, and blood cytometry levels.

### Statistical analysis

All calculations, graphs and statistical analyses were performed using GraphPad Prism software version 8.0. The data were analysed by one-way ANOVA considering the treatments as a single factor. When significant differences were reported by ANOVA, Tukey’s post hoc test was performed for multiple comparisons. p < 0.05 was considered a significant difference. The results are presented as the mean ± SEM.

## Results

The CBZ-treated group and three rats in the *Centella asiatica* 400 mg/kg group did not develop Stage IV or V generalized seizures or SE, so these individuals were not included in the statistical analysis of the behavioural parameters considering that the value of variables was 0. Therefore, whether in the statistical analysis we include values of 0 it could produces an error in the interpretation of the result. However, considering that the statistical analysis of behavioural, biochemical and haematological analyses are independents, those rats were included in this data analysis. When comparing the number of rats that did not undergo SE with pilocarpine administration (30 mg/kg), it was observed that 100% of the CBZ-treated rats and 42.86% of the rats in the *Centella asiatica* 400 mg/kg group did not develop SE. Definitively it is not possible to say that 400 mg/kg of the extract is not effective against SE, considering the modification of the other behavioural variables we could suggest that this dose of the extract attenuated the effects on SE.

### Behavioural evaluation of status epilepticus

**Fig. 1:**
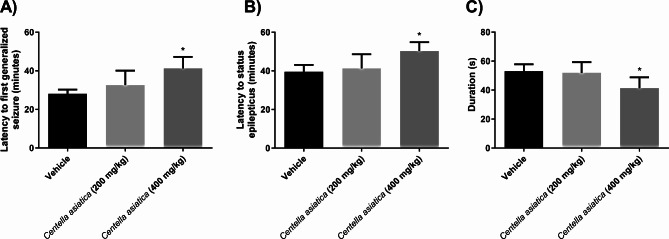
The generation time of the first generalized seizure and epileptic status after the application of pilocarpine. **A**. First generalized seizure, comparison of groups: vehicle vs. *Centella asiatica* 200 mg/kg and 400 mg/kg by one-way ANOVA followed by Tukey’s post hoc test. **B**. SE induction, comparison of the groups: vehicle vs. *Centella asiatica* 200 mg/kg and 400 mg/kg by one-way ANOVA followed by Tukey’s post hoc test. **C**. Duration of the first generalized seizure. One-way ANOVA followed by Tukey’s post hoc test. Data are expressed as the mean ± SEM. * p < 0.05 vs. the vehicle group

Regarding the latency to the first generalized seizure (phase IV or V) and SE induction, significant differences were shown among treatments [F (2, 15) = 6.96, p < 0.0071] and [F (2, 15) = 5.1, p < 0.0204], respectively. The post hoc test revealed that the 400 mg/kg *Centella asiatica* group had a longer latency than that of the vehicle group, while the 200 mg/kg *Centella asiatica* group was devoid of significant differences compared to that of the vehicle group (Fig. 1).

The duration of the first generalized seizure (phase IV or V) revealed significant differences among treatments [F (2, 15) = 4.57, p = 0.0281]. Tukey’s multiple comparison tests showed that the duration of the first generalized seizure in the 400 mg/kg *Centella asiatica* group was shorter than that in the vehicle group, while 200 mg/kg *Centella asiatica* did not change this variable with respect to that of the vehicle group (Fig. 1).

**Fig. 2:**
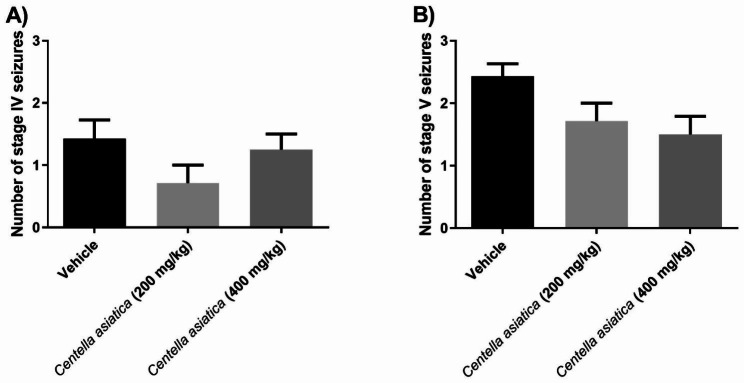
The number of generalized seizures of phase IV **(A)** and phase V **(B)**. Data were analysed with one-way ANOVA. No differences were identified among experimental groups. Data are expressed as the mean ± SEM

The analysis of the number of generalized phase IV and V seizures induced by pilocarpine administration up to one hour after the onset of SE revealed that the number of generalized phase IV seizures was similar among treatments [F (2, 15) = 1.79, p = 0.2007]. No significant differences were found in the number of generalized phase V seizures [F (2, 15) = 3.42, p = 0.0597] considering the treatments (Fig. 2).

### Renal function tests

In the blood chemistry analyses, no significant differences were observed among the treatments, and all values were within the reference ranges: BUN [F (3, 24) = 0.98, p = 0.4634], creatinine [F (3, 24) = 0.59, p = 0.7271], glucose [F (3, 24) = 0.39, p = 0.8730], and urea [F (3, 24) = 1.58, p = 0.4100] (see Table 1).

**Table 1:** Blood chemistry in rats treated with the vehicle, *Centella asiatica* or carbamazepine

Analyte	Vehicle (Purified Water)	*Centella asiatica* (200 mg/Kg)	*Centella asiatica* (400 mg/Kg)	Carbamazepine (300 mg/Kg)	Reference intervals*
**BUN**	6.72 ± 0.16	6.80 ± 0.08	6.71 ± 0.07	6.88 ± 0.04	3.00–7.00 mmol/L
**Creatinine**	21.75 ± 1.45	20.57 ± 1.06	23.14 ± 0.79	18.34 ± 0.99	11.00–28.0 µmol/L
**Glucose**	7.07 ± 0.62	7.64 ± 0.39	7.14 ± 0.41	7.00 ± 0.00	6.00–10.00 mmol/L
**Urea**	18.72 ± 0.21	18.19 ± 0.23	17.57 ± 0.41	18.53 ± 0.34	10.70–20.0 mmol/L

Values are expressed as the mean ± SEM.*Source: Suckow et al., 2006 [47]; Giknis y Clifford, 2008 [48]; Sharp y Villano, 2013 [42]. Rivadeneyra et al., 2018 [49]

### Liver function tests

Analysis of the parameters comprising the liver profile revealed no significant differences among treatments, and values were within the reference range: albumin [F (3, 24) = 1.06, p = 0.4191], ALT [F (3, 24) = 0.90, p = 0.5113], AST [F (3, 24) = 0.42, p = 0.8510], direct bilirubin [F (3, 24) = 0.90, p = 0.5161], indirect bilirubin [F (3, 24) = 0.60, p = 0. 7268], total bilirubin [F (3, 24) = 2.72, p = 0.0462], alkaline phosphatase [F (3, 24) = 2.24, p = 0.0856], glutamyl-transferase [F (3, 24) = 1.05, p = 0.4251], and total proteins [F (3, 24) = 1.21, p = 0.3343] (see Table 2).

**Table 2:** Liver function tests of rats treated with the vehicle, *Centella asiatica* or carbamazepine

Analyte	Vehicle (Purified Water)	*Centella asiatica* (200 mg/Kg)	*Centella asiatica* (400 mg/Kg)	Carbamazepine (300 mg/Kg)	Reference intervals*
**Albumin**	4.40 ± 0.14	4.44 ± 0.08	4.52 ± 0.08	4.45 ± 0.10	4.00–5.00 g/dL
**ALT**	50.24 ± 0.67	44.29 ± 2.93	48.29 ± 1.72	47.43 ± 3.24	19.00–53.00 UI/L
**AST**	153.5 ± 3.22	128.7 ± 8.77	152.4 ± 0.92	106.75 ± 8.29	63.00-157.00 UI/L
**Direct bilirubin**	0.03 ± 0.02	0.03 ± 0.01	0.03 ± 0.01	0.03 ± 0.02	0.03–0.06 mg/dL
**Indirect bilirubin**	0.00 ± 0.01	0.00 ± 0.02	0.01 ± 0.01	0.04 ± 0.02	0-0.10 mg/dL
**Total bilirubin**	0.18 ± 0.10	0.18 ± 0.02	0.18 ± 0.00	0.18 ± 0.00	0.04–0.20 mg/dL
**Alkaline phosphatase**	219.2 ± 31.01	146.6 ± 13.92	205.9 ± 25.79	264.4 ± 14.84	36.00-312.00UI/L
**γ-Glutamyl transferase**	17.26 ± 1.81	18.00 ± 1.63	21.14 ± 0.76	21.71 ± 0.94	8.8–24 UI/L
**Total Protein**	6.20 ± 0.14	6.24 ± 0.12	6.52 ± 0.11	6.28 ± 0.12	5.60–7.60 g/dL

Values are expressed as the mean ± SEM.*Source: Suckow et al., 2006 [47]; Giknis y Clifford, 2008 [48]; Sharp y Villano, 2013 [42]. Rivadeneyra et al., 2018 [49]

### Haematic cytometry

The analysis of the haematic cytometry parameters did not reveal significant differences among the treatments. The results were within the reference intervals: CMHC [F (3, 24) = 0.40, p = 0.8675], erythrocytes [F (3, 24) = 0.15, p = 0.9849], haematocrit [F (3, 24) = 0.52, p = 0.7819], haemoglobin [F (3, 24) = 0.13, p = 0.9908], HCM [F (3, 24) = 0.85, p = 0.5431], leukocytes [F (3, 24) = 2.10, p = 0.1031], platelets [F (3, 24) = 0.85, p = 0.5428] and VCM [F (3, 24) = 1.65, p = 0.1902], and no changes were observed among the groups (Table 3).

**Table 3:** Complete haematic cytometry of rats treated with the vehicle, *Centella asiatica* or carbamazepine

Analyte	Vehicle (Purified Water)	*Centella asiatica* (200 mg/Kg)	*Centella asiatica* (400 mg/Kg)	Carbamazepine (300 mg/Kg)	Reference intervals*
MCHC	31.62 ± 0.26	31.86 ± 0.20	31.80 ± 0.18	31.24 ± 0.18	25.88–32.88 g/dl
Erythrocytes	8.65 ± 0.11	8.57 ± 0.02	8.52 ± 0.04	8.11 ± 0.10	7.8–8.65 mm^3^
Hto	43.09 ± 0.07	45.00 ± 0.00	44.44 ± 0.16	44.26 ± 1.02	35–45%
Hb	15.37 ± 0.61	16.89 ± 0.05	16.59 ± 0.24	16.27 ± 0.25	13.20–17.10 g/dl
MCH	18.26 ± 0.76	19.16 ± 0.22	18.37 ± 0.04	19.49 ± 0.23	15.53–20.05 pg
Leukocytes	12.13 ± 0.18	10.84 ± 0.69	12.64 ± 1.13	11.16 ± 0.57	4.0–17.0 mm^3^
Platelets	722.6 ± 54.28	645.3 ± 31.25	623.1 ± 22.65	717.7 ± 63.54	300.0-1500.0 mm^3^
MCV	56.43 ± 0.79	56.69 ± 0.87	53.57 ± 0.38	54.92 ± 0.72	45–65 fl.

Values are expressed as the mean ± SEM.*Source: Rivadeneyra et al., 2017 [50], 2018 [49]; Zamora-Bello et al., 2022 [51]

### Blood cell count

No significant differences were observed among the treatments with respect to the differential white blood cell counts. All values were within the reference ranges: segmented (mature) neutrophils [F (3, 24) = 0, p = 0.7200], band (immature) neutrophils [F (3, 24) = 1, p = 0.4000], lymphocytes [F (3, 24) = 1, p = 0.2000), eosinophils [F (3, 24) = 0, p = 0.9800), basophils [F (3, 24) = 0, p = 0.4300] and monocytes [F (3, 24) = 2.82, p = 0.6000] (see Table 4).

**Table 4:** Leukocyte differential counts of rats treated with the vehicle, *Centella asiatica* or carbamazepine

Analyte		Vehicle (Purified Water)	*Centella asiatica* (200 mg/Kg)	*Centella asiatica* (400 mg/Kg)	Carbamazepine (300 mg/Kg)	Reference intervals*
Segmented neutrophil	58.00 ± 2.87		60.29 ± 3.20	63.71 ± 4.11	58.00 ± 5.62	35–71%
Band neutrophil	0.57 ± 0.20		0.28 ± 0.18	0.42 ± 0.20	0.14 ± 0.14	0–2%
Lymphocytes	39.71 ± 2.37		33.43 ± 1.80	31.71 ± 1.14	35.71 ± 6.15	20–50%
Eosinophils	0.85 ± 0.40		0.00 ± 0.81	0.85 ± 0.89	1.00 ± 0.43	0–4%
Basophils	0.0		0.28 ± 0.20	0.14 ± 0.14	0.85 ± 0.55	0–1%
Monocytes	2.85 ± 0.26		2.0 ± 0.30	2.85 ± 0.26	4.42 ± 1.08	0–5%

Values are expressed as the mean ± SEM.*Source: Zamora-Bello et al., 2022 [28]

## Discussion

This study evaluated the effect of chronic oral administration of anticonvulsant doses of the *Centella asiatica* standardized extract on biochemical and haematological parameters in SE rats using the lithium-pilocarpine model. The main results included the following: An anticonvulsant effect was shown for chronic oral administration of *Centella asiatica* 400 mg/kg, where approximately half of the group (42.86%) did not present SE. Definitively it is not possible to say that 400 mg/kg of the extract is not effective against SE, considering the modification of the other behavioural variables we could suggest that this dose of the extract attenuated the effects on SE. In addition, experimental subjects who convulsed in this group showed an increased latency to the first generalized seizure and SE and a shorter first seizure duration. Chronic oral administration of *Centella asiatica* did not produce alterations in renal and liver function or in haematic cytometry tests. Therefore, the present results suggest that this compound could be an alternative or therapeutic adjuvant for the treatment of epilepsy that does not cause side effects on the liver and kidneys under the present experimental conditions.

The AED carbamazepine (300 mg/kg p.o.) was used as a pharmacological control. The rats treated with this drug did not present generalized seizures (phase IV or V) or SE. This antiepileptic drug prevents generalized tonic‒clonic seizures and is useful in the treatment of focal-onset or temporal lobe epilepsy, features that are generated in the lithium/pilocarpine model [52–54]. CBZ can be used to prevent the onset or development of generalized tonic-clonic seizures, that occur in SE and the lithium/pilocarpine model [40, 41]. It has also been found that after induction of SE in mice, following injection of kainate into the right dorsal CA1 area of the hippocampus, treatment with CBZ (i.p. 20 and 40 mg/kg) blocks generalized seizures secondary to nonconvulsive SE [55]. However, in another model of kainate with the existence of seizures, it has been reported that the consumption of CBZ-formulated pellets administered in doses of 300 mg/kg completely blocks seizures [56, 57]. It has been reported that 17% of male Wistar rats did not convulse or go into SE with a dose of 30 mg/kg pilocarpine in this model [58]. In contrast, our results showed that a percentage of individuals in the *Centella asiatica* 400 mg/kg group (42.86%) did not experience SE. This finding shows that chronic administration of 400 mg/kg *Centella asiatica* increases latency to the first generalized phase IV/V seizure and EE after pilocarpine injection and in some cases prevents SE from being established. Different studies on epilepsy have suggested that the plant *Centella asiatica* could exert anticonvulsant action by acting on the GABAergic system, as they report that pretreatment administered with this plant at a dose of 200 mg/kg p.o. to rats with SE by PTZ (60 mg/kg, i.p.) mitigates the alterations produced by this model [59]. In this sense, the same research group found that *Centella asiatica* can modulate ATPases (Na^+^/K^+^, Mg^2+^, and Ca^2+^), enzymes that, when inhibited, become a factor that affects neurons, generating epilepsy, which is why they have proposed that it may have anti-epileptic action [60]. On the other hand, administration of 30 mg/kg *Centella asiatica* has also been reported to increase the amount of GABA by modulating the GABA_A_/_B_/benzodiazepine receptor complex [61, 62]. The use of *Centella asiatica* for the treatment of epilepsy has been described in traditional oriental medicine, and although the ethnopharmacological use is reported, clinical studies are lacking to support its chronic use in human epilepsy patients [63, 64]. Consistently, ursolic acid isolated from *Lantana camara* (an antioxidant compound also present in *Centella asiatica*) exerts anticonvulsant effects in the isoniazid seizure model (300 mg/kg i.p.), increasing the latency to first seizure and reducing the seizure duration [65]. In this sense, the pentacyclic triterpenoid compounds (main antioxidant substances in the plant) could be the causal agents of the anticonvulsant effects shown including the increased latency to the first generalized seizure (phase IV or V) and SE, thereby preventing seizures, as well as mitigating the severity of seizures [66]. This idea is reinforced by the fact that these phytochemicals can cross the blood‒brain barrier [67], one of the characteristics that a drug must fulfil to exert its anti-epileptic action [68].

The results of the tests to assess renal function in our study do not show any alterations in the values of creatinine, glucose, urea, and BUN, suggesting that chronic consumption of *Centella asiatica* does not generate risks and/or damage to renal function, so that, under the conditions of the present study, the administration is deemed safe. It has been reported that oral administration of methanolic and ethanolic extracts of *Centella asiatica* produces hypoglycaemic effects at a dose of 250 mg/kg in male Wistar rats with alloxan-induced diabetes [69]. On the other hand, in a model of streptozotocin-induced diabetes in male Sprague‒Dawley rats, *Centella asiatica* has been reported to have hypoglycaemic effects at oral doses of 500 and 1000 mg/kg. In contrast, our results do not show a decrease in blood glucose levels, which may be because the hypoglycaemic effect of *Centella asiatica* is only observed in animals with diabetes, as it promotes the correct functioning of insulin receptors to internalize glucose into the cells, whereas in healthy animals, as in our situation, there is no observable effect because glucose uptake is already occurring correctly [70]. It has also been reported that in conditions of unilateral urethral obstruction in Sprague‒Dawley rats, subchronic intragastric administration of *Centella asiatica* at doses of 56 and 168 mg/kg does not generate alterations in creatinine or BUN [71]. Furthermore, the literature reports that in rats with diabetic nephropathy treated with aqueous extracts of the plant, creatinine levels are maintained at normal levels, as it improves renal pathology [72]. The studies cited were carried out in populations with induced diseases; in contrast, this work used experimental subjects who were healthy throughout the administration period, so hypoglycaemic effects were not exhibited.

The results obtained from the liver function tests in the present study showed no alterations in the parameters described above so that under the conditions of the present study, chronic consumption of *Centella asiatica* does not present any adverse reactions and does not generate hepatotoxicity, making it safe to consume. In this way, it has been reported that the aminotransferases ALT and AST, which were elevated in rats with streptozotocin-induced diabetes, returned to their baseline values, suggesting that this effect is a consequence of the improvement in insulin action caused by the administration of *Centella asiatica* [70]. On the other hand, subchronic oral administration of *Centella asiatica* in male Wistar rats with hepatotoxicity caused by antituberculosis drugs has been reported to have a hepatoprotective effect by normalizing high levels of bilirubin and alkaline phosphatase [73]. In another study using male Sprague‒Dawley rats, *Centella asiatica* was found to have a hepatoprotective effect, decreasing the levels of ALT, AST, alkaline phosphatase, and total bilirubin, which were elevated due to dimethylnitrosamine-induced liver injury, and the total protein and albumin values were not altered in any way [74]. Although the literature reports that the use of *Centella asiatica* decreases the concentrations of several parameters of liver function tests, these effects were not observed in the present work, as the hepatoprotective effects are not exhibited in healthy experimental subjects, as mentioned by Chivapat et al., 2011 [75] and Deshpande et al., 2015 [76], who reported that the use of *Centella asiatica* for up to 90 days does not alter ALT, AST, albumin, total bilirubin and total protein values [75, 76].

Concerning the various parameters associated with haematic cytometry, chronic oral administration of *Centella asiatica* did not modify these values, and blood smears showed that there were no alterations in morphology, nor were there any changes observed in the percentages of the cell populations of white blood cells, as they were all within the reference intervals. In this regard, a study in which male Wistar rats were chronically administered standardized extracts of *Centella asiatica* at doses of 10, 100, and 1000 mg/kg [75] coincides with the present study with respect to blood cytometry, since the erythrocyte count, haemoglobin, MCH, MCHC, MCV, platelet count and leukocyte count reported are within our reference ranges used in this study [49, 50, 77]. Furthermore, according to their blood cell count, *Centella asiatica* did not cause alterations in leukocyte percentages in the control group (administered only water), which is consistent with this study [75]. Accordingly, another study also showed that haematic cytometry and blood cell counts were not altered by chronic consumption of *Centella asiatica* in male Sprague‒Dawley rats [76]. Similar results concerning haematic cytometry were found in a study carried out in 2019, in which haematological values were evaluated in albino mice after subacute administration of different doses of *Centella asiatica* (200, 400, 800, and 2000 mg/kg) in an ethanolic vehicle. It was shown that none of the doses altered the haematic cytometry values [78]. Although several studies have reported that there are no alterations in the parameters constituting the haematic cytometry and blood cell counts by the consumption of *Centella asiatica*, compared to the present study, the percentages of lymphocytes and neutrophils differ from those reported, in contrast to those observed by Chivapat et al., 2011 [75] and Deshpande et al., 2015 [76]. This situation could be explained by that fact that each research laboratory or vivarium has different conditions for sanitary maintenance such that the experimental subjects could have an unobservable infection during their handling, which would activate the immune system, modifying the percentages of the different white blood cells [76].

## Conclusions

Oral administration of *Centella asiatica* L. Urb extract for 35 consecutive days showed anticonvulsant effects without generating nephrotoxicity or hepatotoxicity and did not cause alterations in blood cytometry. These findings suggest that *Centella asiatica* L. could serve as an alternative to ameliorate seizures in epileptic patients without altering the biochemical levels of liver and kidney function tests.

## Data Availability

The datasets used and/or analysed during the current study are available from the corresponding author on reasonable request.

## Abbreviations

AEDs: antiepileptic agents
ALT: alanine aminotransferase
ANOVA: analysis of variance
AST: aspartate aminotransferase
BUN: blood urea nitrogen
Ca^2+^: calcium
CBZ: carbamazepine
EDTA: ethylenediaminetetraacetic acid
GABA: gamma aminobutyric acid
GSH: reduced glutathione
GSH-Px: glutathione peroxidase
Hb: haemoglobin
Hto: haematocrit
IP: intraperitoneal
K^+^: potassium
LC3: light chain microtubule-binding protein 3
LiCl: lithium chloride
MCH: mean corpuscular haemoglobin
MCHC: mean corpuscular haemoglobin concentration
MCV: mean corpuscular volume
MDA: malondialdehyde
Mg^2+^: magnesium
Na^+^: sodium
P.O.: Per os
PTZ: pentylenetetrazole
ROS: reactive oxygen species
SC: subcutaneous
SE: status epilepticus
SEM: standard error of the mean
SOD: superoxide dismutase
